# Variable thermal expansion of glass-ceramics containing Ba_1−x_Sr_x_Zn_2_Si_2_O_7_

**DOI:** 10.1038/s41598-017-03132-x

**Published:** 2017-06-13

**Authors:** Christian Thieme, Martin Schlesier, Eze Oji Dike, Christian Rüssel

**Affiliations:** Otto-Schott-Institut für Materialforschung, Jena University, Fraunhoferstr. 6, 07743 Jena, Germany

## Abstract

Up to now, the thermal expansion behavior of multiphase glass-ceramics cannot be predicted reliably because of the nescience about the formation of the type and concentration of crystalline phases. In the system BaO-SrO-ZnO-SiO_2_, recently a new phase based on Ba_1−x_Sr_x_Zn_2_Si_2_O_7_ solid solutions was found, which exhibits unexpected low and highly anisotropic thermal expansion, which can be used for an adjustment of the thermal expansion properties. In the case of sealing materials for high-temperature reactors, the formation of this phase should be avoided. Hence, in this manuscript the concentration thresholds in which these solid solutions precipitate from glasses were determined. The phase analysis was correlated with the thermal expansion behavior of the glass-ceramics. Depending on the Ba/Sr-ratio of the glasses and the considered temperature range, the coefficients of thermal expansion of the glass-ceramics vary between 19.4·10^−6^ K^−1^ and 4.8·10^−6^ K^−1^. The concentration thresholds in which the as mentioned phases form via crystallization of glasses differ strongly from the literature values obtained via conventional ceramic mixed oxide route.

## Introduction

The thermal expansion of alkaline earth containing silicate or borosilicate glasses and glass-ceramics can be adjusted to high values and is hence suitable for sealing applications, where the glass-ceramic seal is in contact to high thermal expansion metals, such as Crofer or Nicrofer or ceramics, such as zirconia^1–6^. One of the most popular examples, in which glass-ceramics are needed in order to seal different materials, is the solid oxide fuel cell (SOFC)^7^. Here, the coefficient of thermal expansion (CTE) has to be adjusted precisely in order to avoid excessive stresses at the interface to the sealed materials^8^.

Glass-ceramics based on alkaline earth silicates possess high thermal expansion, which strongly depends on the composition of the base glass^9^. Typical CTE values of such materials are in the range from 8 to 12·10^−6^ K^−1^, according to the CTEs of the respective crystalline phases^7, 10^. However, even higher values up to above 17·10^−6^ K^−1^ can be obtained by the precipitation of BaZn_2_Si_2_O_7_ and isostructural solid solutions, in which Zn^2+^ is partially replaced by other bivalent cations of similar ionic radii^11, 12^. The thermal expansion behavior of BaZn_2_Si_2_O_7_ is almost temperature independent and very high below a phase transition at around 280 °C^13^. Above the phase transition temperature, very low and temperature dependent thermal expansion can be observed^14^. Recently, it was reported that the CTEs of glass-ceramics in the base system BaO-ZnO-SiO_2_ decrease drastically if SrO is added to the glass composition due to the formation of Ba_1−x_Sr_x_Zn_2_Si_2_O_7_ solid solutions with the crystal structure of the high-temperature polymorph of BaZn_2_Si_2_O_7_ (HT-BaZn_2_Si_2_O_7_)^9, 15^. These solid solutions exhibit very low thermal expansion and hence sealing glasses with much lower CTEs can be obtained^9, 16^. However, these glasses cannot be used in high-temperature reactors such as the SOFC because of the high CTE mismatch between the seal and other components of the cell^7^. Especially the crystallization of phases with very high and very low thermal expansion in one and the same glass-ceramic material should be avoided in order to guarantee long-term stability during thermal cycling and good mechanical properties of the seals.

Furthermore, orthorhombic Ba_1−x_Sr_x_Zn_2_Si_2_O_7_-crystals with the structure of HT-BaZn_2_Si_2_O_7_ show highly anisotropic thermal expansion behavior, this is negative thermal expansion in the direction of the lattice parameter b and positive thermal expansion in the direction of the lattice parameters a and c, whereas the c parameter has a much higher thermal expansion than the a parameter. The origin of the thermal expansion is described in detail in ref. 15 but can shortly be described by a stretching movement of ZnO_4_-chains in the direction of the lattice parameter c, causing very high thermal expansion. Furthermore, these tetrahedra become strongly compressed, which leads to a contraction in the b direction. This contraction is extremely strong and hence, the mean value of the coefficient of thermal expansion is negative.

Solid solutions of the composition Ba_1−x_Sr_x_Zn_2_Si_2_O_7_ crystallize in high concentrations from appropriate glass compositions^17^. The phase stability range of the pure crystalline materials was investigated in ref. 14. At x < 0.1, phases with the crystal structure of LT-BaZn_2_Si_2_O_7_ as well as mixtures of LT- and HT-BaZn_2_Si_2_O_7_ form. At higher values of x, the HT-phase is stable up to around x = 0.9, a threshold above which single phase ceramic cannot be obtained anymore^14, 18^. However, there are hints which indicate that both, the HT- as well as the LT-phase, also form outside of the given phase stability region, if precipitated from glasses^9^.

The aim of this study is to clarify the effect of the substitution of Ba^2+^ by Sr^2+^ on the phase formation of glass-ceramics, in which Ba_1−x_Sr_x_Zn_2_Si_2_O_7_ solid solutions occur as the main phase. In these glasses, the Ba/Sr-ratio was varied and the phase formation and its effect on the thermal expansion were investigated.

### Experimental Procedure

Glasses of 200 g were melted in Pt crucibles at 1400 °C for 2 h using an inductively heated furnace. The batches were prepared from reagent grade raw materials: BaCO_3_ (>98.5%, Merck), SrCO_3_ (pure, VEB Laborchemie Apolda), ZnO (>99%, Carl Roth GmbH & Co. KG), SiO_2_ (>99%, Carl Roth GmbH & Co. KG), ZrO_2_ (>99%, Carl Roth GmbH & Co. KG), La_2_O_3_ · H_2_O (pure, VEB Laborchemie Apolda) and H_3_BO_3_ (>99%, Merck). After casting the glasses on a copper block, they were transferred into a furnace preheated to 700 °C, which was switched off in order to allow the glass to cool down slowly.

From the glasses, which were cooled down to room temperature, cylindrically shaped samples were drilled out in order to measure the thermal expansion behavior of the glasses. For this purpose, a NETZSCH Dil 402 PC with a heating rate of 5 K/min was used. The CTEs were determined as the slopes (linear regressions) of the thermal expansion curves between 200 and 500 °C. Furthermore, the bulk glasses were used to measure the density using a helium pycnometer MICROMERITICS AccuPyc 1330.

From the bulk glasses, powders were prepared by crushing the glass to a grain size <400 µm using a steel mortar. This powder was then transferred into a mortar mill and sieved to a grain size <71 µm. This powder was used for the DSC (differential scanning calorimetry), HSM (side-view hot-stage microscopy) and XRD (X-ray diffraction) measurements. The DSC was performed up to 1100 °C using a LINSEIS DSC Pt-1600 and a heating rate of 10 K/min. The HSM was performed using a self-built device, where the samples were placed in a heated corundum tube next to a thermocouple. With an increment of 2 K, images of the projection of the sample were saved in order to illustrate the sintering behavior. The evaluation was performed by the determination of the area of this projection.

The glass powder was also heat treated in order to study the formation of the appearing crystalline phases. The sintered and crystallized powders were afterwards milled again and XRD was performed in a 2θ-range from 10° to 60° using a RIGAKU MiniFlex 300.

From these diffractograms, the lattice parameters and the phase content was determined using the Rietveld software MAUD (Materials Analysis Using Diffraction)^19^. Therefore, the data from the crystallographic data from the literature or the Inorganic Crystal Structure Database (ICSD) of Zn_2_SiO_4_ (ref. 20, ICSD 2425), SiO_2_ (ref. 21, ICSD 83849), and Sr_2_ZnSi_2_O_7_ (ref. 22, ICSD 247476) were taken. In the case of LT-BaZn_2_Si_2_O_7_, the dataset of the isostructural compound BaCo_2_Si_2_O_7_ (ref. 23, ICSD 81473) was taken. Phases with the structure of HT-BaZn_2_Si_2_O_7_ were refined using the data reported in ref. 15, which have the composition Ba_0.6_Sr_0.4_Zn_2_Si_2_O_7_.

The thermal expansion of the glass-ceramics was also studied by dilatometry as described above. However, instead of bulk samples, glass powders were used, which were pressed into cylindrical shape and afterwards the respective heat treatment was performed. From the dilatometric measurements, so called “technical CTE values” were calculated using the following equation: α_T1-T0_ = (L_1_ − L_0_)/(L_0_ · (T_1_ − T_0_)), where L_0_ and L_1_ are the samples lengths at the temperatures T_0_ and T_1_, respectively.

Chemical analyses of the as casted glasses were performed with a Jeol JSM 7001 F electron microscope equipped with an energy dispersive X-ray spectroscopy (EDS) instrument. Quantification was performed with an acceleration voltage of 30 kV evaluating the following emission lines: Zn L, Si K, Ba L, Sr K, Zr L, and La L.

## Results

Transparent and homogenous glasses were obtained after the melting procedure. No crystalline phases could be detected with XRD. The respective diffractograms are given as Supplementary Figure S1. The compositions in which only the Ba/Sr-ratio was varied are summarized in Table 1 in units of [mol%] together with the respective sample names. In order to show that there is no large difference between the batch composition and the real chemical composition, quantification was performed applying EDS on the samples Sr-00, Sr-05, and Sr-11.

**Table 1:** Batch compositions of the glasses in mol%. The chemical compositions of the samples Sr-00, Sr-05, and Sr-11 were also determined with EDS.

Sample name	batch/EDS	BaO	SrO	ZnO	SiO_2_	ZrO_2_	B_2_O_3_	La_2_O_3_
Sr-00	batch	16	-	35	45	1	2	1
	EDS	16.6	0.5	35.6	45.4	0.9	*	1.1
Sr-01	batch	15	1	35	45	1	2	1
Sr-02	batch	14	2	35	45	1	2	1
Sr-03	batch	13	3	35	45	1	2	1
Sr-04	batch	12	4	35	45	1	2	1
Sr-05	batch	11	5	35	45	1	2	1
	EDS	11.4	6.3	35.3	45.3	0.6	*	1.1
Sr-06	batch	10	6	35	45	1	2	1
Sr-07	batch	9	7	35	45	1	2	1
Sr-08	batch	8	8	35	45	1	2	1
Sr-09	batch	7	9	35	45	1	2	1
Sr-10	batch	6	10	35	45	1	2	1
Sr-11	batch	5	11	35	45	1	2	1
	EDS	5.0	12.9	34.6	46.2	0.3	*	1.0
Sr-12	batch	4	12	35	45	1	2	1
Sr-13	batch	3	13	35	45	1	2	1
Sr-14	batch	2	14	35	45	1	2	1
Sr-15	batch	1	15	35	45	1	2	1
Sr-16	batch	—	16	35	45	1	2	1

*Detected, but not quantified.

**Figure 1:**
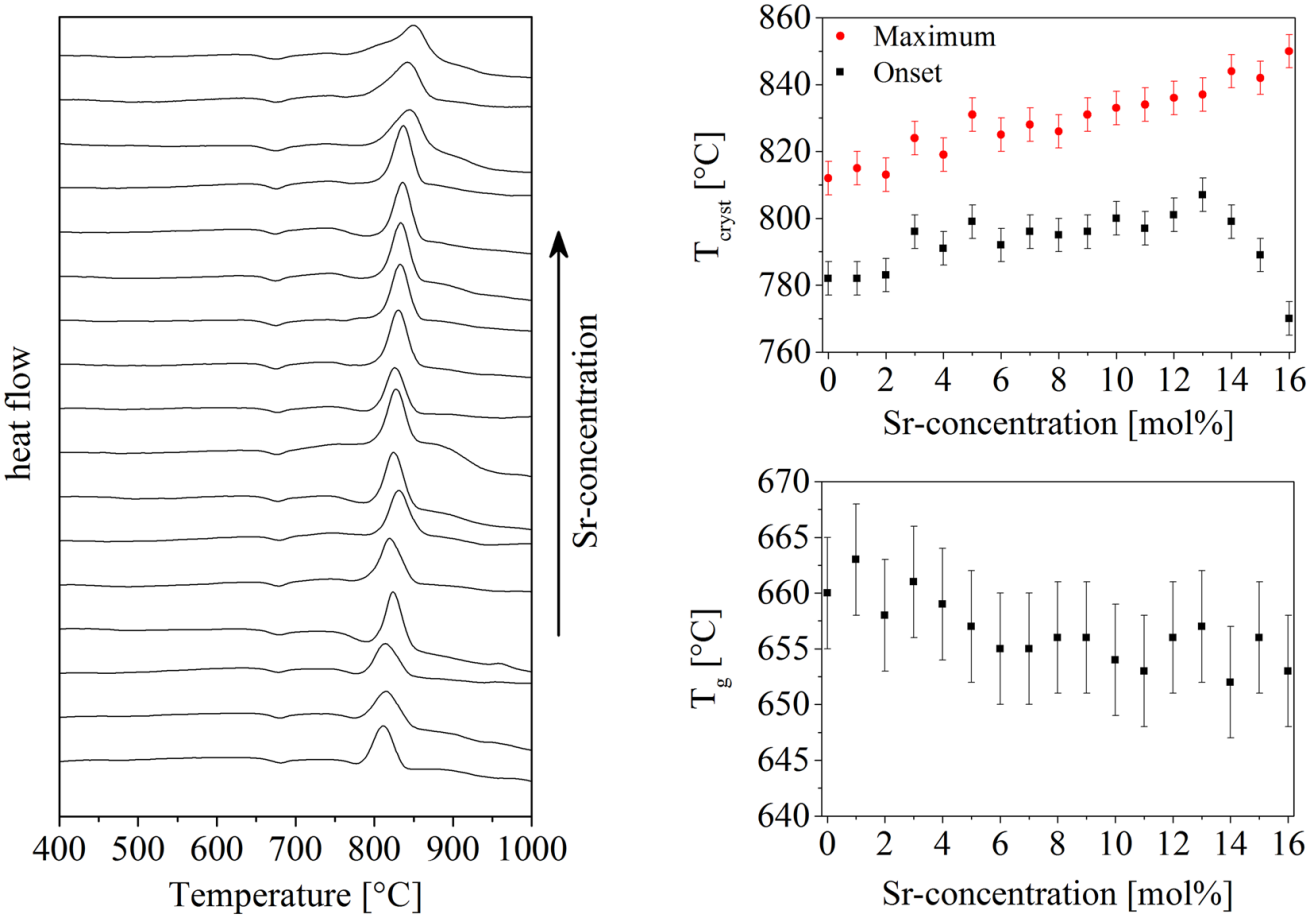
Thermal analysis of the glasses. On the left, DSC-curves of all samples are illustrated. The upper right graph shows the change of the crystallization temperature as a function of the Sr-concentration. The lower right part shows the glass transition temperature determined via dilatometry in dependence of the Sr-concentration.

The DSC-curves of all 17 glass compositions are illustrated in Fig. 1 (left part), where fine grained powder (<71 µm) was characterized. It can be seen that the glass transition temperatures (T_g_) remain almost constant, whereas the crystallization peaks are shifted to higher temperatures with increasing Sr-concentration. In the lower right part of Fig. 1, the glass transition temperatures are illustrated as a function of the Sr-concentration of the glasses. Furthermore, the T_g_-values from both, dilatometry and DSC, are summarized in Table 2 together with other characteristic properties from DSC, dilatometry, HSM and pycnometry. The T_g_ values are in the range from 649 to 661 °C. The measuring error of these values is around ± 3 K. A steady decrease in T_g_ with increasing SrO-concentration is observed, nevertheless, the effect is fairly small. In the upper right part of Fig. 1, the crystallization temperatures (onset and maximum) are displayed as a function of the Sr-concentration of the glass. The maximum of the crystallization peak increases almost linearly, whereas the onset temperatures increase from the sample Sr-00 to Sr-13. At higher Sr-concentrations, the onset is shifted to lower temperatures. This can also be seen in Fig. 1 (left), where the shape of the crystallization peak changes at high Sr-concentrations, i. e. a shoulder appears at lower temperatures, which indicates the formation of at least two phases.

**Table 2:** Properties of the glasses determined with pycnometry (pyc), dilatometry (dil), DSC, and hot-stage microscopy (HSM). T_g_ - onset of the glass transition; T_d_ - dilatometric softening temperature; α_300–500_ - technical coefficient of thermal expansion between 300 and 500 °C; α_reg_ - coefficient of thermal expansion determined by the slope of a linear fit of the dilatation curve between 200 and 500 °C; T_FS_ - sintering onset (first shrinkage); T_MS_ - end of sintering (maximum shrinkage). The values of the sample Sr-08 are reported in ref. 17.

Sample	Density [g/cm³]	T_g_ [°C]	T_g_ [°C]	T_d_ [°C]	α_300–500_ [10^−6^·K^−1^]	α_reg_ [10^−6^·K^−1^]	T_FS_ [°C]	T_MS_ [°C]
Method	pyc	DSC	dil	dil	dil	dil	HSM	HSM
Sr-00	4.105	653	660	699	7.66	7.54	716	762
Sr-01	4.079	655	663	702	7.76	7.58	721	765
Sr-02	4.090	653	658	701	7.74	7.57	719	771
Sr-03	4.062	658	661	706	7.98	7.72	721	777
Sr-04	4.050	656	659	699	7.65	7.54	722	766
Sr-05	4.045	655	657	693	7.85	7.64	715	769
Sr-06	4.026	652	655	704	7.68	7.51	721	764
Sr-07	4.005	655	655	702	7.77	7.60	722	770
Sr-09	3.981	653	656	696	7.92	7.71	710	770
Sr−10	3.960	649	654	700	7.32	7.16	716	774
Sr-11	3.956	654	653	699	6.98	6.80	710	772
Sr-12	3.942	654	656	695	7.51	7.32	710	770
Sr-13	3.919	652	657	704	7.45	7.29	725	771
Sr-14	3.906	649	652	688	7.15	6.88	715	766
Sr-15	3.871	649	656	703	7.58	7.44	725	770
Sr-16	3.854	647	653	692	7.71	7.52	709	765

**Figure 2:**
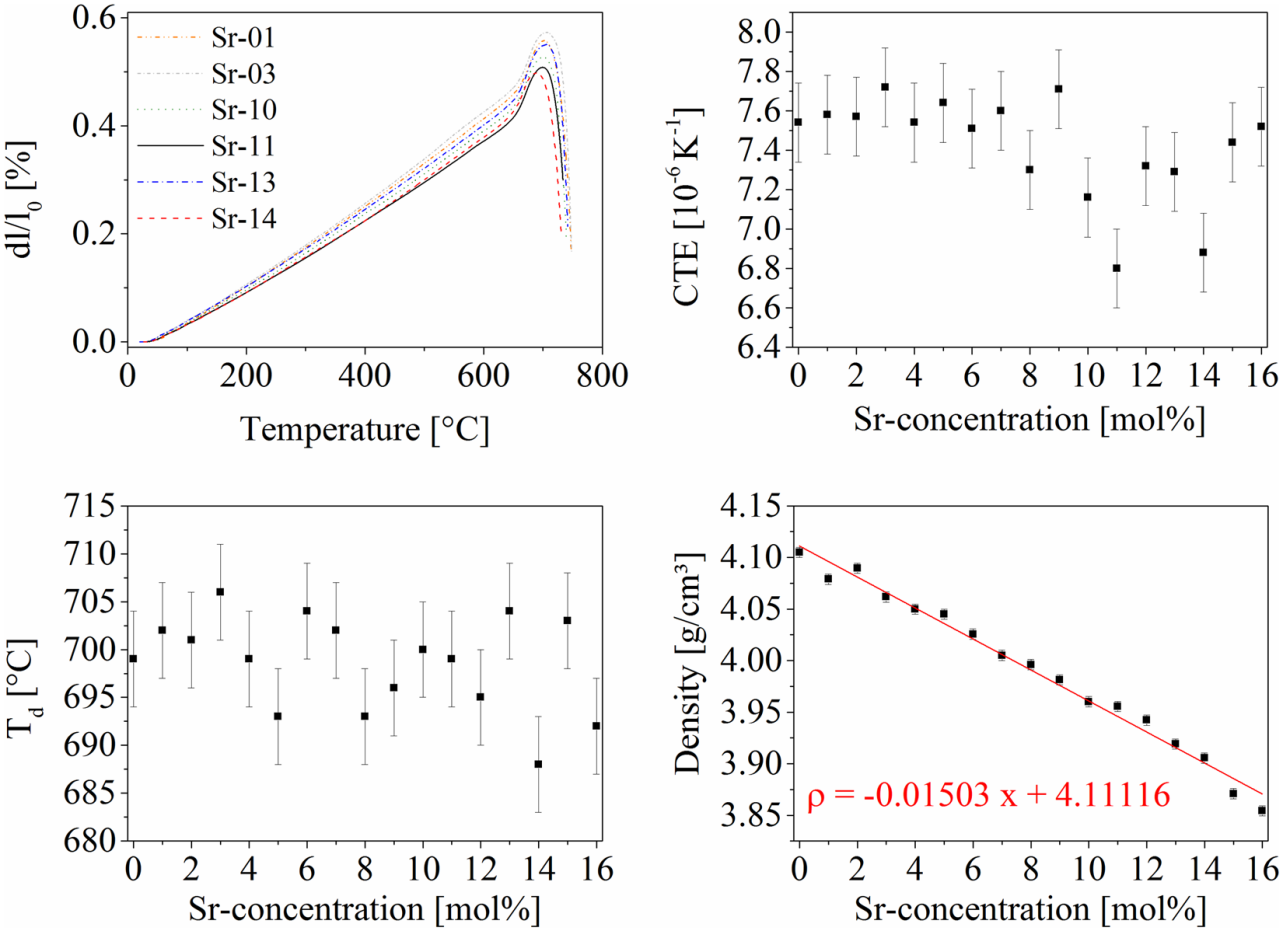
Thermal analysis and pycnometric results. On the upper left, dilatometric curves of elevated samples are displayed. The upper right shows the coefficients of thermal expansion as a function of the Sr-concentration. The lower left Figure displays the dilatometric softening temperature as a function of the Sr-concentration. The lower right diagram shows the density in dependence of the Sr-concentration. The density was fitted by a linear regression. The respective formula is given inside the diagram, where x is the respective Sr-concentration in units of [mol%].

The dilatometric curves of bulk glass samples are illustrated in the upper left part of Fig. 2 for elevated samples. The CTEs as well as the T_d_-values (T_d_ = dilatometric softening temperatures) are also illustrated in Fig. 2, where no clear trend of these values can be observed. The CTEs are all in the range from 6.8 to 8.0 10^−6^ K^−1^ and show a slight decrease of the CTEs with increasing Sr-concentration and a reincrease of these values at high Sr-concentrations. The density (lower right part of Fig. 2) decreases linearly with increasing Sr-concentration. The solid line is a regression line, the attributed regression parameters are given inside the graph, where x is the Sr-concentration in units of [mol%].

**Figure 3:**
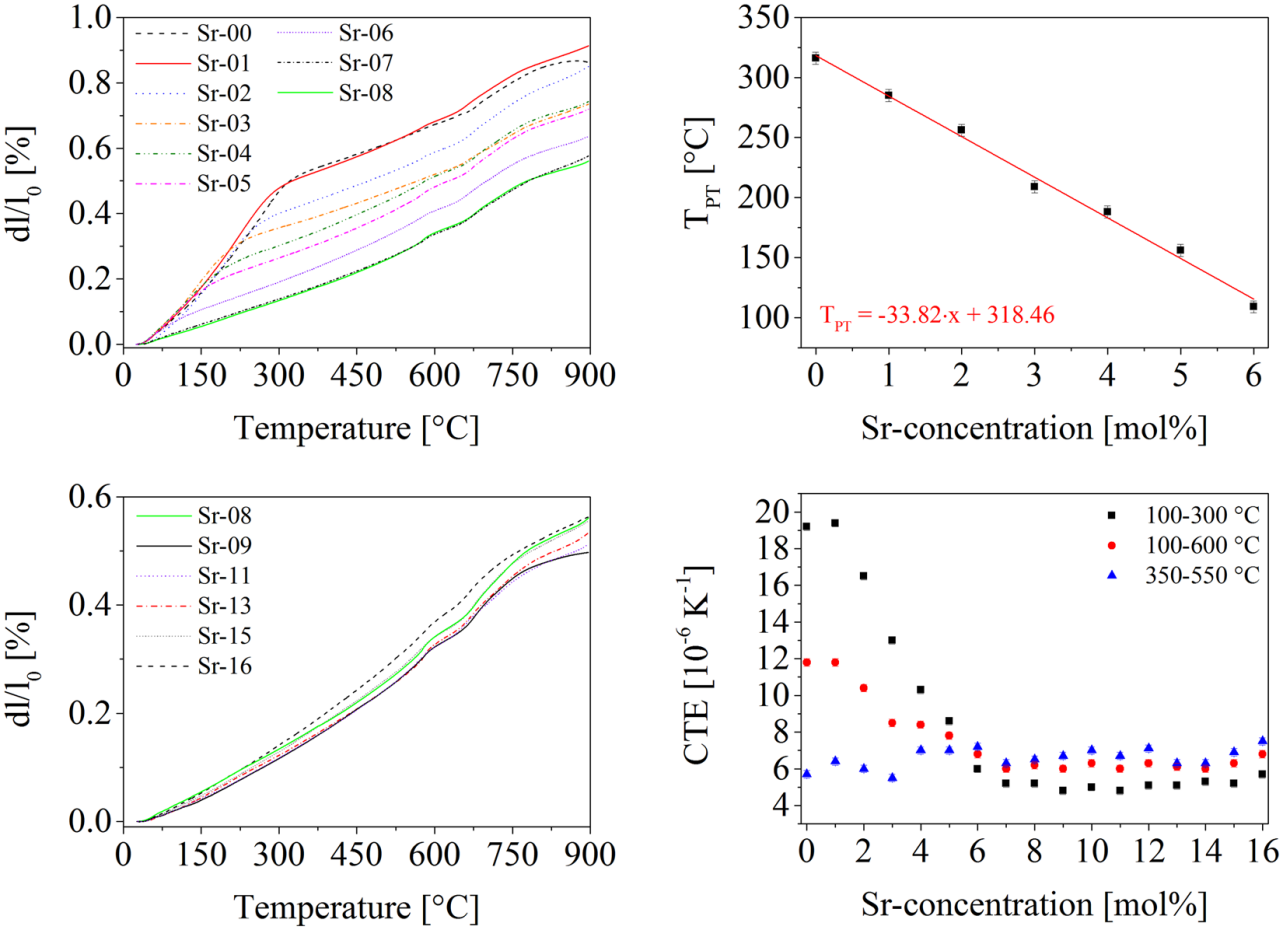
Results from dilatometry of pressed powder compacts, which were sintered and crystallized at 900 °C for 1 h. The upper and the lower left diagrams show the dilatation curves for different compositions. The upper right part shows the phase transition temperatures (T_PT_) of the transition from phases with the structure of LT-BaZn_2_Si_2_O_7_ to HT-BaZn_2_Si_2_O_7_. The linear regression is given inside the diagram, where the phase transition temperature in units of [°C] is given as a function of the Sr-concentration x in units of [mol%]. The lower right part shows technical coefficients of thermal expansion in dependence of the Sr-concentration calculated for different temperature ranges.

In comparison to the dilatometric thermal expansion of the glasses, the respective glass-ceramics show a strong dependence of the thermal expansion behavior as a function of the composition. The dilatometric curves recorded from glass-powder sintered at 900 °C for 1 h are shown in Fig. 3. In the upper left part, the thermal expansion curves of samples with Sr-concentrations between 0 and 8 mol% are shown. A kink is observed at temperatures between room temperature and around 300 °C, which can be attributed to the phase transition of LT-BaZn_2_Si_2_O_7_ to its HT-polymorph. In the pure BaZn_2_Si_2_O_7_-phase, this transition takes place at 280 °C. These phase transition temperatures decrease linearly as a function of the Sr-concentration (see upper right part of Fig. 3). The linear regression of the phase transition temperatures as a function of the Sr-concentration (denoted as “x”) is given inside the graph. The kink cannot be observed at Sr-concentrations higher than 7 mol%, i. e. if around 45% of the BaO is substituted with SrO. Furthermore, a small volume jump can be observed at around 570 °C, which can clearly be seen in the case of higher Sr-concentration. The temperature of this kink does not change with the composition of the samples. At a Sr-concentration below 3 mol%, this effect is notably less pronounced. At around 650 °C, another kink can be observed, which is also at the same position for all samples.

The CTEs decrease with increasing Sr-concentration. The CTEs also exhibit a strong dependence on the temperature and therefore, the CTEs given in the lower right part of Fig. 3 are calculated for different temperature ranges. In the range from 100 to 300 °C, the samples Sr-00 and Sr-01 exhibit very high CTEs of 19.2 and 19.4·10^−6^ K^−1^, respectively. At higher Sr-concentrations, the CTEs decrease and reach a minimum value of 4.8·10^−6^ K^−1^ for the samples Sr-09 and Sr-11. At high Sr-concentrations, a slight re-increase of the CTE is observed.

A similar behavior can be found in the case that the CTEs were calculated in the range from 100 to 600 °C. Furthermore, CTEs were determined at temperatures above the phase transition but below the kink at 570 °C. For this purpose, CTEs from 350 to 550 °C were calculated, however, they do not show a clear trend.

**Figure 4:**
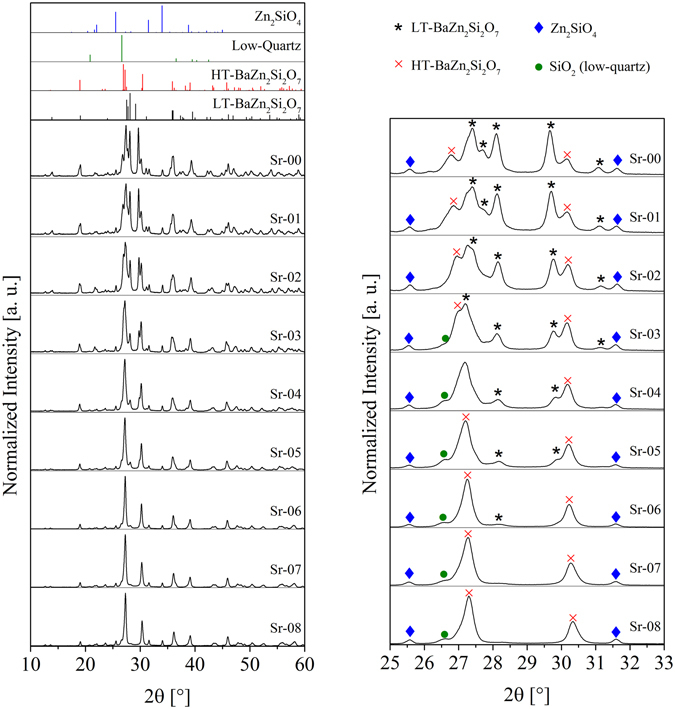
X-ray diffraction patterns of samples crystallized at 900 °C for 1 h with Sr-concentrations in the range from 0 to 8 mol%. On the left side, the full measuring range is illustrated together with the theoretical peak positions calculated from the respective ICSD entries or from the literature (LT-BaZn_2_Si_2_O_7_^24^, HT-BaZn_2_Si_2_O_7_^15^, Zn_2_SiO_4_ (willemite)^20^, SiO_2_ (low-quartz)^21^). On the right side, the same patterns are illustrated in a narrower 2θ-range from 25–33° and the peaks are attributed to certain crystalline phases.

**Figure 5:**
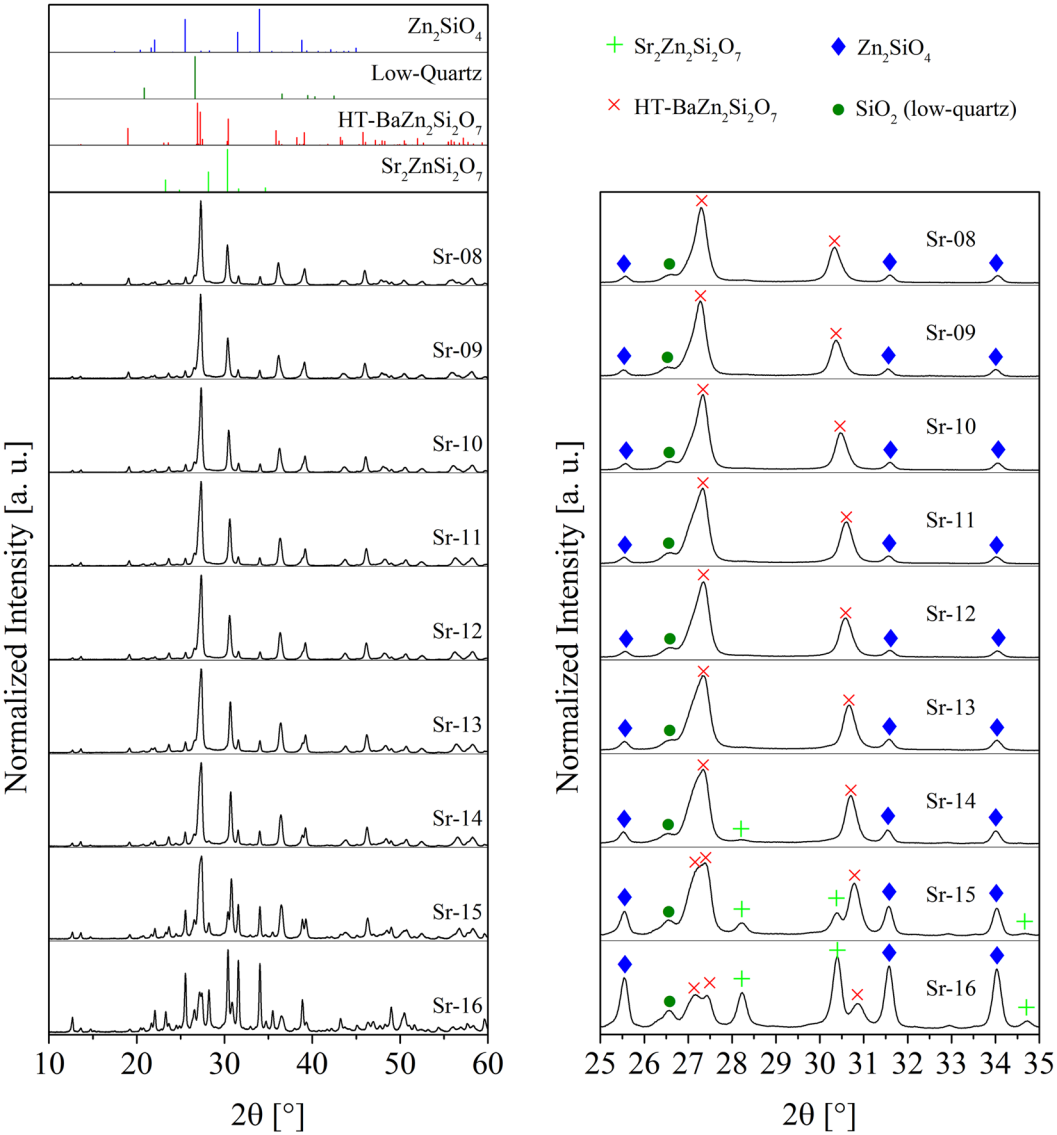
X-ray diffraction patterns of samples crystallized at 900 °C for 1 h with Sr-concentrations in the range from 8 to 16 mol%. On the left side, the full measuring range is illustrated together with the theoretical peak positions calculated from the respective ICSD entries or from the literature (HT-BaZn_2_Si_2_O_7_^15^, Zn_2_SiO_4_ (willemite)^20^, SiO_2_ (low-quartz)^21^, Sr_2_ZnSi_2_O_7_^22^). On the right side, the same patterns are illustrated in a narrower 2θ-range from 25–35° and the peaks are attributed to certain crystalline phases.

**Figure 6:**
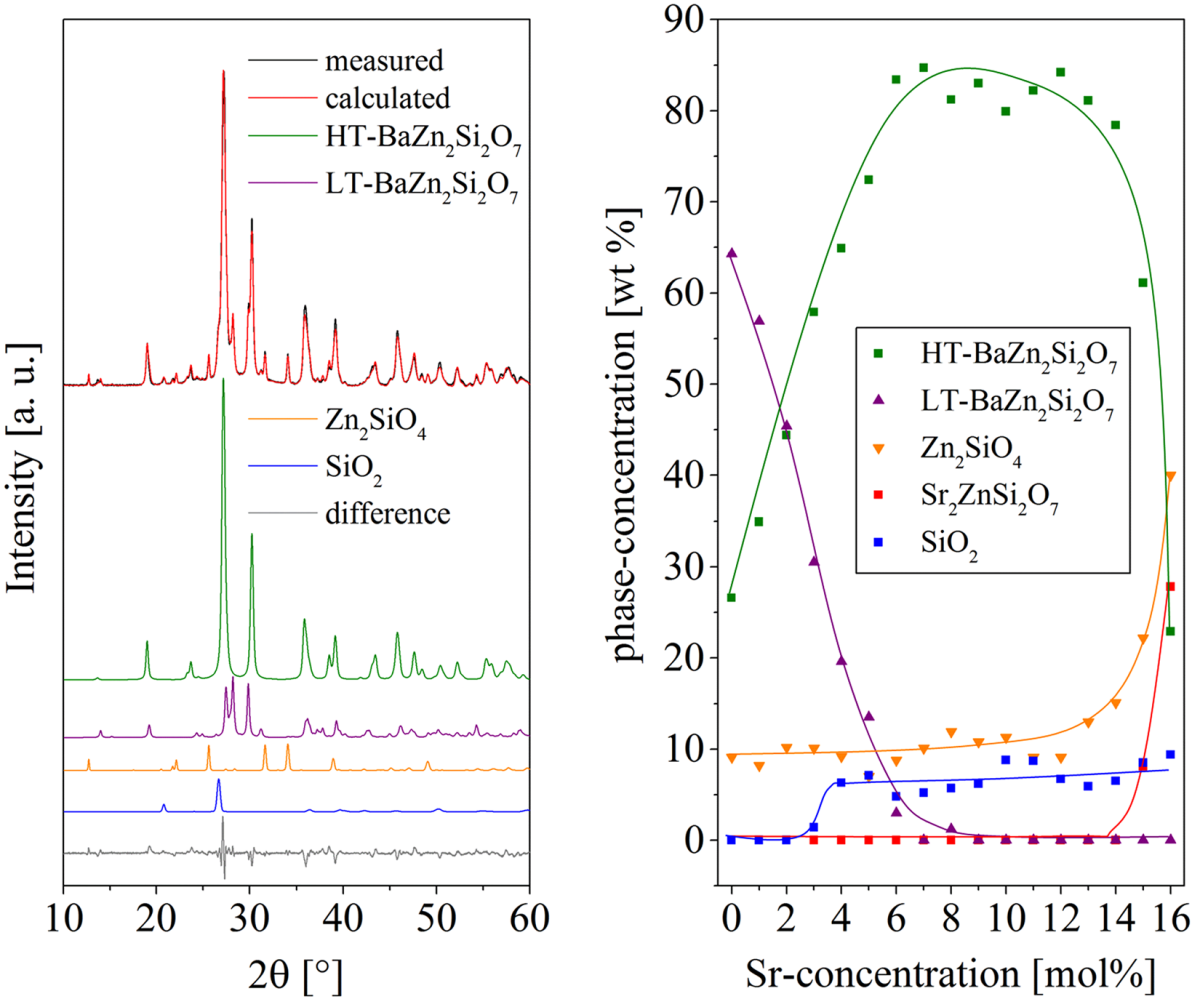
Results from quantitative phase analysis. On the left side, the result from the refinement of the sample Sr-04 heat treated at 900 °C for 1 h is shown. On the right side, the concentration of the crystal phases is given as a function of the Sr-concentration of the glasses. The sum of all crystalline phases is 100%, i. e. the residual amorphous phase is not considered in this illustration. The inserted lines are just a guide for the eyes. Uncertainties are around ± 5 mol%.

The phase formation at 900 °C can be seen in Fig. 4 for the samples with 0 to 8 mol% SrO and in Fig. 5 from 8 to 16 mol% of SrO. The peaks can reliably be attributed to the phases Zn_2_SiO_4_ (willemite), SiO_2_ (low-quartz) as well as Ba_1−x_Sr_x_Zn_2_Si_2_O_7_ solid solutions, which can have both, the crystal structure of HT- as well as LT-BaZn_2_Si_2_O_7_. From the diffractograms, the concentrations of the phases inside the glass-ceramics were calculated (see Fig. 6). The Sr-free glass shows a mixture of phases with the crystal structure of LT- as well as HT-BaZn_2_Si_2_O_7_. Furthermore, around 10% of Zn_2_SiO_4_ (willemite) have been formed. Up to around 3% of SrO, almost no low-quartz was detected. At higher temperatures, 5 to 10% SiO_2_ were found. However, the amount of SiO_2_ is highly erroneous, because only one or two peaks can be observed within the diffractograms and the major peak is more or less overlapped by the phase with the structure of HT-BaZn_2_Si_2_O_7_. A further increase of the Sr-concentration leads to a decreasing amount of LT-BaZn_2_Si_2_O_7_, which can no longer be observed in notable quantities if the Sr-concentration is higher than 6%. Between 6 and 13 mol% of SrO, the main phase is a solid solution with the structure of HT-BaZn_2_Si_2_O_7_ with around 80 to 85 wt%. The other components are willemite and low-quartz. Furthermore, a small amount of residual glassy phase should also be found in all glass-ceramics containing B_2_O_3_, ZrO_2_ and La_2_O_3_, which are not incorporated in a crystalline phase. The glassy phase was not quantitatively characterized. Above around 13 mol% of SrO, the amount of HT-BaZn_2_Si_2_O_7_ solid solutions decreases drastically from above 80 to below 30%. This decrease runs parallel to an increase in the concentration of willemite as well as Sr_2_ZnSi_2_O_7_. The latter starts to form if the SrO concentration is at least 14 mol% (Sr-14).

**Figure 7:**
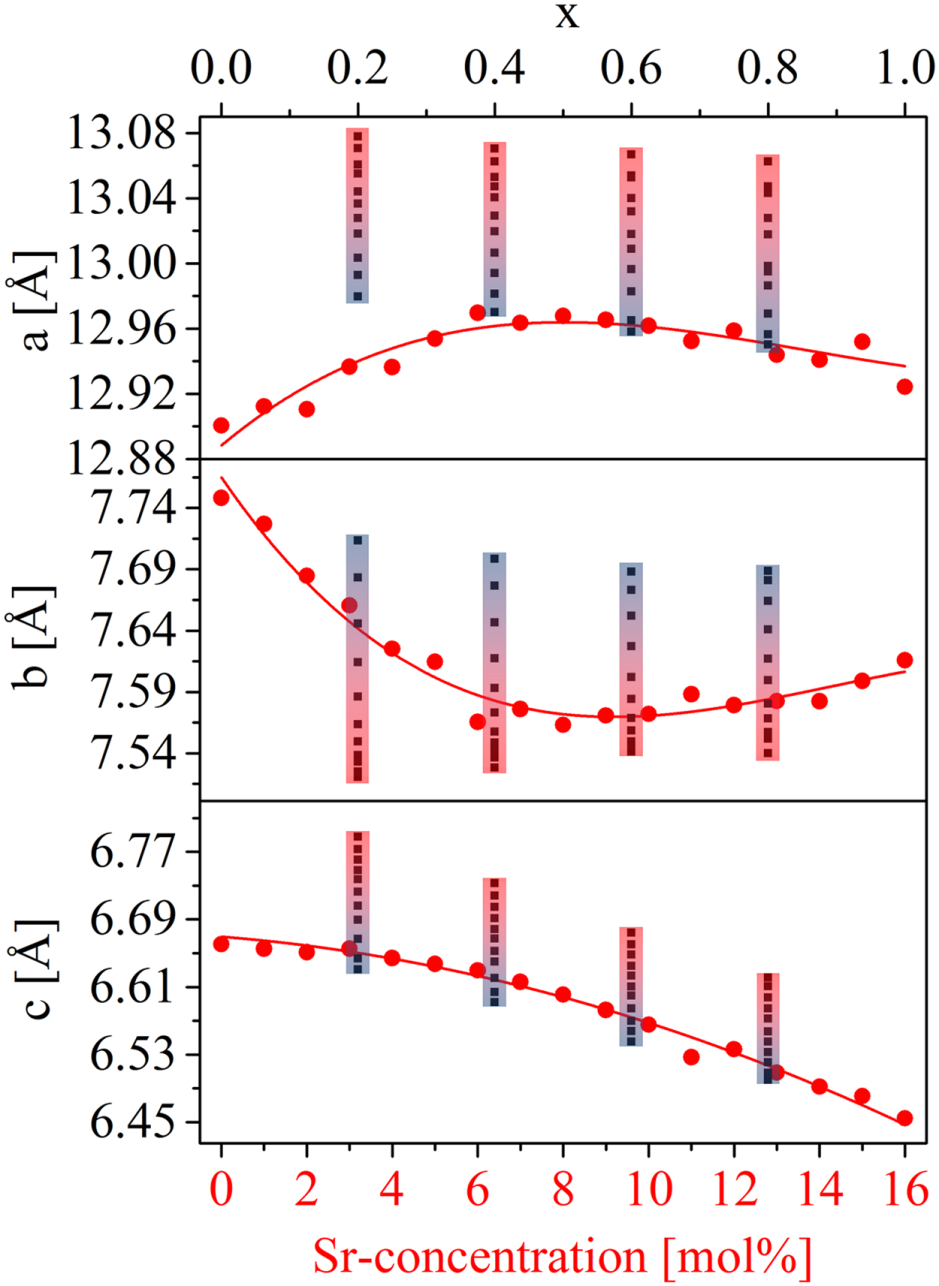
Lattice parameters of Ba_1−x_Sr_x_Zn_2_Si_2_O_7_ solid solution phases with the crystal structure of HT-BaZn_2_Si_2_O_7_. The red circles belong to the lattice parameters determined from the glass-ceramics with different Sr-concentrations heat treated at 900 °C for 1 h. The black squares belong to the lattice parameters determined in ref. 14 at room temperature and between 100 and 1000 °C in steps of 100 K. The temperature increase is marked by the colored rectangles, where the temperature increases from blue to red. These values belong to the upper x-axis. Both x-axes are the same in the case that all alkaline earth ions are solely incorporated into the Ba_1−x_Sr_x_Zn_2_Si_2_O_7_ solid solutions.

From the refinement of the crystal phases, also the lattice parameters were determined, which is illustrated in Fig. 7 for the crystals with the structure of HT-BaZn_2_Si_2_O_7_ as a function of the Sr-concentration inside the starting glass compositions. The measured lattice parameters are shown as red dots. The a parameter increases with increasing Sr-concentration of the starting glass composition up to around 8 mol% Sr. At higher Sr-concentrations, the lattice parameter a decreases slightly. By contrast, the b parameter decreases between 0 and 8 mol% Sr and reincreases from 8 to 16 mol%. Lattice parameter c decreases continuously, but not linearly.

Besides the measured lattice parameters, Fig. 7 also shows the lattice parameters reported in ref. 14 as squares. The latter values were determined at different temperatures (30, 100, 200, 300, 400, 500, 600, 700, 800, 900, and 1000 °C), which is indicated by the colored bars (blue color means low temperatures and red color means high temperatures). These literature values were determined with stoichiometric single phase powders of the general composition Ba_1−x_Sr_x_Zn_2_Si_2_O_7_ and the upper x-axes should be used. In the case of glass-ceramics in which all alkaline earth ions are incorporated into a phase with the structure of HT-BaZn_2_Si_2_O_7_, the x-value of the upper x-axis can be directly converted into the Sr-concentration of the lower x-axis. However, this is only valid in the case of Sr-concentrations from around 7 to 13 mol%, i. e. the lattice parameters of the phases with x = 0.6 (Ba_0.4_Sr_0.6_Zn_2_Si_2_O_7_) and x = 0.8 (Ba_0.2_Sr_0.8_Zn_2_Si_2_O_7_) prepared via solid state reaction as reported in ref. 14 can directly be compared with those of the phases precipitated from the glasses containing 9–10 and 12–13 mol% of Sr. In this concentration range, the lattice parameter a determined from the precipitated phase is in good agreement with the lattice parameters of the pure phases determined between room temperature and 100 °C. In the case of the b parameter, the values obtained from the glass-ceramics are comparable with those of the single phase powders at 600 to 700 °C. The c parameters have almost the same values if compared to the literature values determined at around 300 °C.

Also the lattice parameters of LT-BaZn_2_Si_2_O_7_ show a distinct trend between 0 and 6 mol% Sr (see Supplementary Figure S2). At higher Sr-concentrations, this phase cannot be detected anymore. The lattice parameters of willemite and low-quartz are scattering and show no clear trend.

## Discussion

As summarized in Table 1, the compositions of the final glasses and the batch compositions are in good agreement. However, a reliable quantification of B_2_O_3_ is not possible with EDS and this is also the most volatile species in these glasses. B_2_O_3_ was hence not quantified, but it was detected with EDS. The role of B_2_O_3_ in these glasses is only to enlarge the glass forming region. A huge influence of the B_2_O_3_ on the thermal expansion properties of the final crystallized samples is not expected by the authors.

The results of the thermal analyses of the glasses in Figs 1 and 2 clearly show that the thermal properties of the glasses such as T_g_ and T_d_ as well as the CTEs are almost not affected by the substitution of BaO with SrO. As expected, the density decreases linearly with decreasing BaO-concentration. The compositions of the glasses were not determined experimentally because in the past similar glasses were produced in almost the same way and their compositions were quantified and showed no significant variation from the batch composition^11, 12, 17^.

The crystallization behavior changes significantly if BaO is substituted by SrO. First hints can be found in the DSC-curves (see Fig. 1), where the crystallization onset is shifted to lower temperatures if the Sr-concentration exceeds 13 mol%. A shoulder appears which should be due to the formation of Sr_2_ZnSi_2_O_7_ proved via XRD. Below 14 mol% SrO, the DSC signal does not change significantly with the exception that the crystallization peaks are shifted to higher temperatures with increasing Sr-concentrations. However, the appearing crystalline phases and hence also the thermal expansion behavior changes significantly. The sample without SrO, this is the sample Sr-00, shows a thermal expansion curve divided into two main parts, one with very high thermal expansion at lower temperatures and one with a lower thermal expansion at higher temperatures. The origin of the change of the thermal expansion from high to low is the phase transition from LT- to HT-BaZn_2_Si_2_O_7_ as already reported in ref. 12. This phase transition is shifted linearly to lower temperatures with increasing Sr-concentration as illustrated in the upper right corner of Fig. 3. Interestingly, even at a SrO-concentration of 6 mol%, this is a substitution of 37.5% of BaO with SrO, the phase transition can be observed above room temperature. Hence, in samples with more than 6 mol% of SrO, almost no crystalline phases with the structure of the LT-phase of BaZn_2_Si_2_O_7_ can be observed anymore.

This concentration threshold differs strongly from that obtained in ref. 14, where pure stoichiometric ceramics were characterized. There, it is reported that single phase solid solutions with the crystal structure of HT-BaZn_2_Si_2_O_7_ start forming if 10 to 20 mol% of BaO are substituted by SrO. Hence, in comparison to stoichiometric crystalline ceramics, glass-ceramics need a higher amount of SrO in order to obtain solely the HT-phase.

Furthermore, in the case of the samples Sr-14, Sr-15, and Sr-16, Sr_2_ZnSi_2_O_7_ is additionally formed and hence fractions of the alkaline earth oxides are also incorporated in this phase. Possibly, also minor concentrations of BaO are incorporated into this phase, but this was not proven here and could also not reliably be verified via XRD. In the case of pure crystalline ceramics of the compositions Ba_1−x_Sr_x_Zn_2_Si_2_O_7_, the HT-phase is stable up to x = 0.9, this is a substitution of 90% of BaO by SrO^14^. At higher SrO-concentrations, no single phase materials can be obtained anymore. In the case of glass-ceramics, this behavior starts with the sample Sr-14 and becomes stronger for Sr-15 and Sr-16. In the sample Sr-14, 87.5% of BaO are replaced by SrO and hence, the upper SrO-concentration thresholds are almost identical in the case of glass-ceramics and single phase ceramics.

The appearing phases are illustrated in Figs 4 and 5, where it is seen that besides the HT- and LT-phase of BaZn_2_Si_2_O_7_ also small willemite and low-quartz concentrations are found. The appearance of low-quartz was also confirmed by the very slight volume jump at around 570 to 575 °C observed in the dilatometric curves, which should be due to the low-quartz/high-quartz phase transition. The formation of the latter is due to the composition of the glasses, which has a somewhat higher SiO_2_ concentration than a stoichiometric ceramic material. The quartz concentration remains almost constant for all glass compositions (see Fig. 6). At SrO-concentrations below 4 mol%, the quantitative analysis of low-quartz obtained from the XRD-patterns in Figs 4 and 5 is highly erroneous because there is a strong overlapping of the main peak of low-quartz and the main peak of HT-BaZn_2_Si_2_O_7_ solid solutions. However, a very small volume jump at around 570 °C can be observed in the respective dilatometric curves, which is an indication of the presence of low-quartz also in the samples Sr-00, Sr-01, Sr-02, and Sr-03. Hence, the kink in the line of SiO_2_ in Fig. 6 between 2 and 4% might not be as pronounced. The quantitative phase analysis also shows that the willemite concentration is around 10% and increases at higher SrO-concentrations, which runs parallel with the increasing formation of Sr_2_ZnSi_2_O_7_. The occurrence of willemite as well as of quartz is in agreement with the glass composition which in relation to Ba_1−x_Sr_x_Zn_2_Si_2_O_7_ has an excess in ZnO as well as in SiO_2_.

The results from quantitative phase analyses of samples crystallized at 900 °C are in agreement with the thermal expansion behavior between 100 and 300 °C where the maximum values of CTE for the samples Sr-00 and Sr-01 and the lowest values between Sr-07 and Sr-13 were observed. The extremely high thermal expansion at low SrO-concentrations is due to the precipitation of LT-BaZn_2_Si_2_O_7_ and the re-increase of the CTE at higher SrO-concentrations is caused by the formation of Sr_2_ZnSi_2_O_7_, with a CTE of around 9.5·10^−6^ K^−1^ ^9^. The CTEs strongly depend on the temperature and hence the CTEs in the lower right part of Fig. 3 are given for different temperature ranges. The values from 350 to 500 °C are measured above the phase transition temperature of phases with the structure of LT-BaZn_2_Si_2_O_7_ and below the temperature of the volume jump of low-quartz. In this range, the thermal expansion of all the samples is almost independent of temperature. The lowest value was measured for the sample Sr-03 (5.5·10^−6^ K^−1^) and the highest for the sample Sr-16 (7.5·10^−6^ K^−1^).

In Fig. 7, the lattice parameters of Ba_1−x_Sr_x_Zn_2_Si_2_O_7_ solid solutions with the structure of HT-BaZn_2_Si_2_O_7_ are illustrated for both, glass-ceramics as well as stoichiometric ceramics. As already mentioned, the lattice parameters from the stoichiometric material at x = 0.6 and 0.8 can be compared to the lattice parameters of the glass-ceramics between 9 and 13 mol% of SrO. Especially the b parameter shows a strong deviation if both, the glass-ceramic and the stoichiometric material are compared. The values of the glass-ceramics are much lower than in the case of the stoichiometric material, which should be due to the stresses inside the samples forming during cooling. Under the assumption that the glass-ceramics are stress-free at the crystallization temperature of 900 °C, the following should happen during cooling concerning the lattice parameters of the HT-phase of BaZn_2_Si_2_O_7_. The crystallographic a axis contracts together with the other appearing phases and is almost not affected. The lattice parameter b strongly expands and gets compressed because of the surrounding phases, which all contract during cooling. The c parameter strongly contracts during heating, i. e. it contracts much more than the surrounding phases and therefore, this parameter is higher than in a stress-free case.

The lowered b-parameter, which has around the same value as a stress-free material at around 600 to 700 °C might increase the CTE of this phase, which was also experimentally proven via *in-situ* XRD in ref. 17. The reason should be that this parameter is compressed so strongly that a further contraction during heating is not possible anymore. Especially an adjustment of the glass composition should solve this problem. Therefore, the amount of phases with the structure of HT-BaZn_2_Si_2_O_7_ should be increased and the precipitation of high thermal expansion phases such as quartz should be hindered and as a result, the CTE of the glass-ceramic material should be lowered.

## Conclusion

The thermal expansion behavior of glass-ceramics from the base system BaO-SrO-ZnO-SiO_2_ can be varied between 19.4 and 4.8·10^−6^ K^−1^ only by the variation of the Ba/Sr-ratio. The change in the thermal expansion behavior is attributed to the formation of phases with the crystal structure of LT- and HT-BaZn_2_Si_2_O_7_. If around 45 to 90 mol% of BaO are replaced by SrO, all the alkaline earth ions are incorporated into solid solutions with the structure of HT-BaZn_2_Si_2_O_7_. This concentration range is somewhat smaller than in the case of pure single phase ceramics. However, in this range very low thermal expansion can be expected. Furthermore, the lattice parameters of this HT-phase differ from those reported on single phase ceramics. Especially the lattice parameter b is much smaller in the case of the glass-ceramics reported here, which is due to a compression inside the direction in which negative thermal expansion occurs. This effect together with the formation of low-quartz and willemite leads to CTEs above 4·10^−6^ K^−1^.

### Data availability statement

The datasets generated during and/or analyzed during the current study are available from the corresponding author on reasonable request.

## Electronic supplementary material

Supplementary Dataset 1
