# Supplementary material for: Variable thermal expansion of glass-ceramics containing Ba1−xSrxZn2Si2O7
Source: Sci Rep. 2017 Jun 13;7:3344. doi: 10.1038/s41598-017-03132-x (PMC5469781; doi:10.1038/s41598-017-03132-x)
Supplement: Supplementary file 1 — Supplementary Dataset 1 [file 41598_2017_3132_MOESM1_ESM.doc]

**Variable thermal expansion of glass-ceramics containing Ba_1-x_Sr_x_Zn_2_Si_2_O_7_**

Christian Thieme, Martin Schlesier, Eze Oji Dike, Christian Rüssel

Otto-Schott-Institut für Materialforschung, Jena University, Fraunhoferstr. 6, 07743 Jena


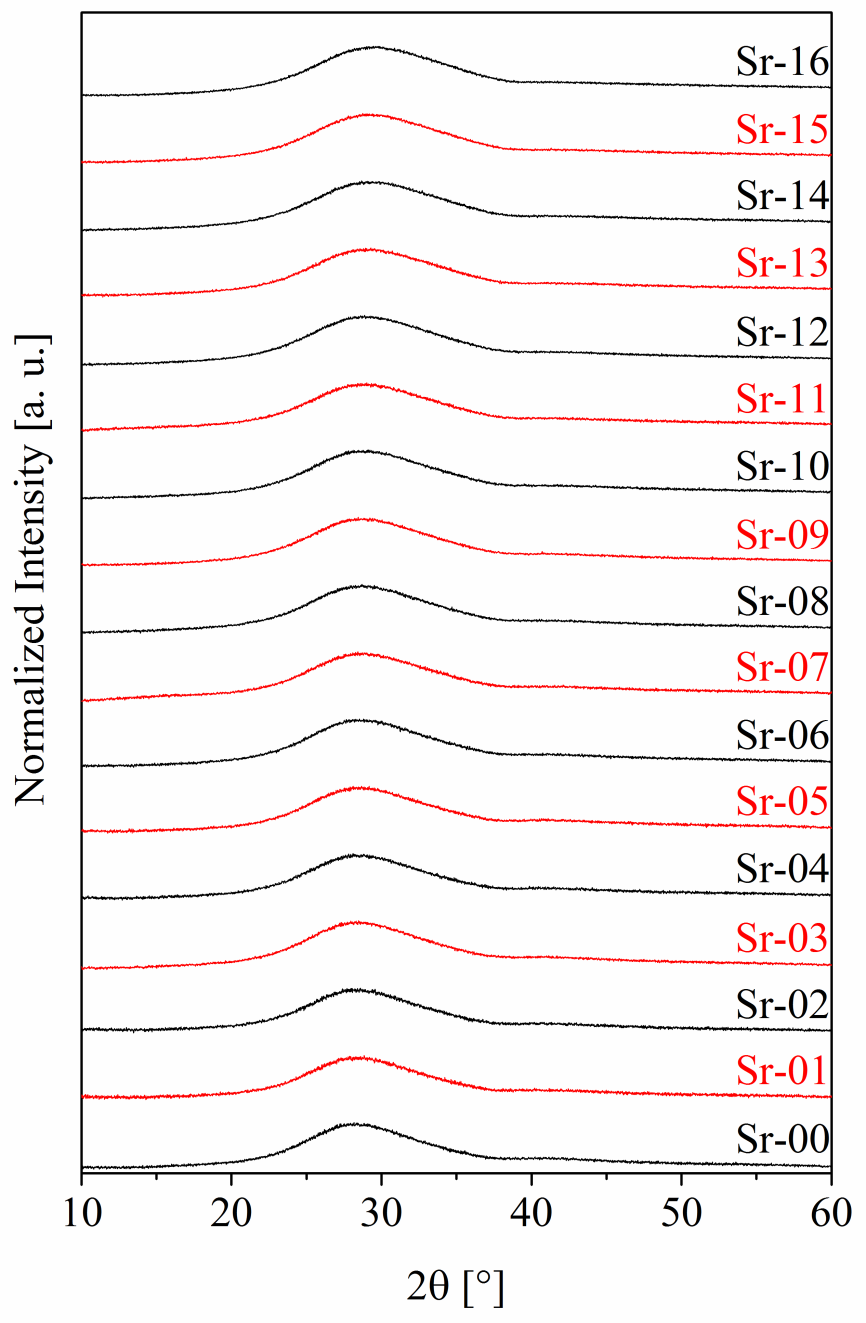


**Figure S1:** XRD patterns of the glasses.


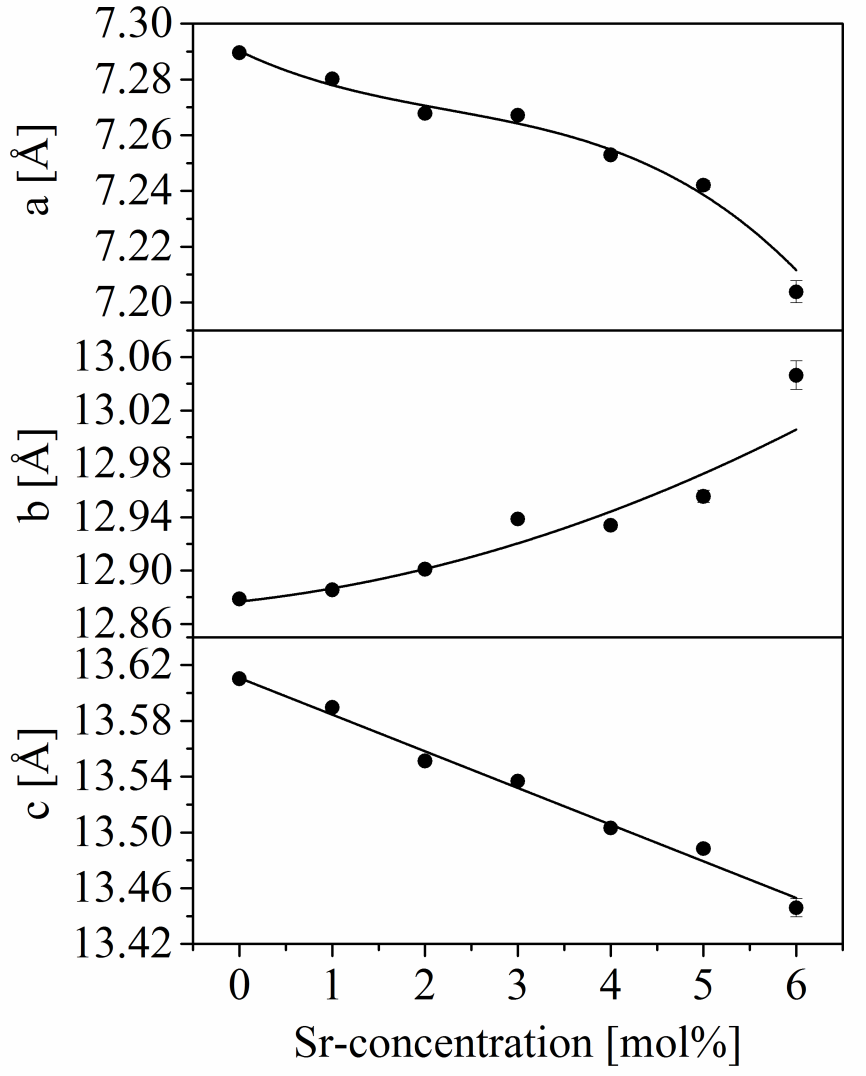


**Figure S2:** Lattice parameters of Ba_1-x_Sr_x_Zn_2_Si_2_O_7_ solid solution phases with the crystal structure of LT-BaZn_2_Si_2_O_7_. The lattice parameters were determined from the glass-ceramics with different Sr-concentrations heat treated at 900 °C for 1 h.
